# Dr. G. Allpress Simmons

**Published:** 1914-08-08

**Authors:** 


### Obituary.

Dr. G. Allpress Simmons, the *x*-ray authority, has died in London, aged forty-nine. A St. Mary's Hospital man, he qualified in 1890, and became an M.D. of London University in 1892. It was not until 1897, previous to which house appointments at his hospital and private practice had occupied his time, that he began to study *x*-ray therapeutics, but he soon became medical officer in charge of the *x*-ray department at St. Mary's Hospital. Subsequently he became senior radiographer at St. George's.

